# The value of serum IL-4 to predict the survival of MDS patients

**DOI:** 10.1186/s40001-022-00948-w

**Published:** 2023-01-04

**Authors:** Zhaoyun Liu, Xintong Xu, Likun Zheng, Kai Ding, Chun Yang, Jincheng Huang, Rong Fu

**Affiliations:** Department of Hematology, Tianjin Medical University General Hospital, Tianjin, 300052 China

**Keywords:** Myelodysplastic syndromes, Immune index, Cytokines, Prognosis

## Abstract

**Background:**

Immune indicators are routinely used for the detection of myelodysplastic syndrome (MDS), but these are not utilized as a reference indicator to assess prognosis in MDS-related prognostic evaluation systems, such as the World Health Organizational prognostic scoring system, the international prostate symptom score, and the revised international prostate symptom score.

**Methods:**

We examined immune indicators, including cluster of differentiation (CD)3, CD4, CD8, CD56, CD19, interleukin (IL)-2, IL-4, IL-6, IL-10, tumor necrosis factor-a, and interferon-γ in 155 newly diagnosed MDS patients. We also conducted a correlation analysis with clinical indices.

**Results:**

IL-4 was found to be a predictor of survival in these 155 patients using the receiver operating characteristic curve, with 5.155 as the cut-off point. Patients with serum IL-4 levels ≥ 5.155 had a lower overall survival (OS) than those with IL-45.155 at diagnosis. Furthermore, multivariate analysis revealed that IL-4 levels > 5.155 were an independent predictor of OS (hazard ratio: 0.237; 95% confidence interval, 0.114–0.779; *P* = 0.013). In addition, serum IL-4 expression in the three different scoring systems showed significant differences in the survival of medium- to high-risk MDS patients (*P* = 0.014, *P* < 0.001, *P* < 0.001).

**Conclusions:**

According to our study, IL-4 levels at the time of diagnosis can predict MDS prognosis in patients as a simple index reflecting host systemic immunity.

**Supplementary Information:**

The online version contains supplementary material available at 10.1186/s40001-022-00948-w.

## Background

Myelodysplastic syndrome (MDS) is a clonal disease that originates from hematopoietic stem cells. It is characterized by low hematopoietic function, dysplasia and peripheral blood cytopenia [1, 2]. Abnormal cloned cells exhibit impaired differentiation and maturation in the bone marrow, pathological or ineffective hematopoiesis, and a high risk of transformation into acute myeloid leukemia. Different clinical treatments are chosen for patients with varying levels of risk. Increased apoptosis of hematopoietic precursors is a key feature of MDS [3, 4]. This phenomenon may be influenced by both genetic and epigenetic alterations [5], as well as immune mechanisms. Some studies have suggested that the interaction between malignant clones and the bone marrow microenvironment causes clonal expansion and apoptotic responses in MDS [6]. Various bone marrow stromal cells work collaboratively in the bone marrow niche, regulating the differentiation, development, and self-renewal of bone marrow hematopoietic stem cells and maintaining a dynamic balance in the hematopoietic microenvironment [7–9]. These stromal cells include endothelial cells, macrophages, adipocytes, fibroblasts, osteoblasts, and chondrocytes [10]. Autocrine production of angiogenic molecules is associated with the self-renewal of bone marrow monocyte precursors and promotes the production of oncogenic cytokines [3]. These cells can regulate hematopoiesis by secreting cytokines. Both the innate and adaptive immune systems are active in the ecological niche of MDS and play an important role in its pathogenesis [11].

The World Health Organization prognostic scoring system (WPSS), International Prostate Symptom Score (IPSS-R), and Revised International Prostate Symptom Score (IPSS-R) can be used to evaluate the prognosis of MDS patients by monitoring age, sex, neutrophil count, hemoglobin, platelet count, cytopenia series, bone marrow primitive cell ratio, and karyotype, as well as predict the risk grade of MDS patients and propose related treatment strategies [12–15]. The percentage of blasts, the number of cytopenic lines, and the cytogenetic indications of bone marrow, are all included in the classification criteria, but immune indicators prognosis are not. In MDS patients with different types (RA/RARS/RCMD/RAEB/RAEB-T), the ratio of Th1/Th2 will vary to different degrees. However, there is no relevant research on the correlation between the expression levels of Th1/Th2 immune indicators and the prognosis of MDS patients with various risk grades [16–18]. In our study, we focused on MDS-associated TB cell subsets and cytokine assay results, with the goal of identifying a specific immune indicator. Following identification, we investigated the variation in this indicator in newly diagnosed MDS patients, and subsequently examining the influence of its content on the survival of MDS patients. Given the significant differences in serum IL-4 levels between low-risk and medium- to high-risk patients, it may be used as an independent risk factor for predicting MDS patient survival. Moreover, this index may be included in the prognostic scoring system.

## Materials and methods

### Patients

**Fig. 1:**
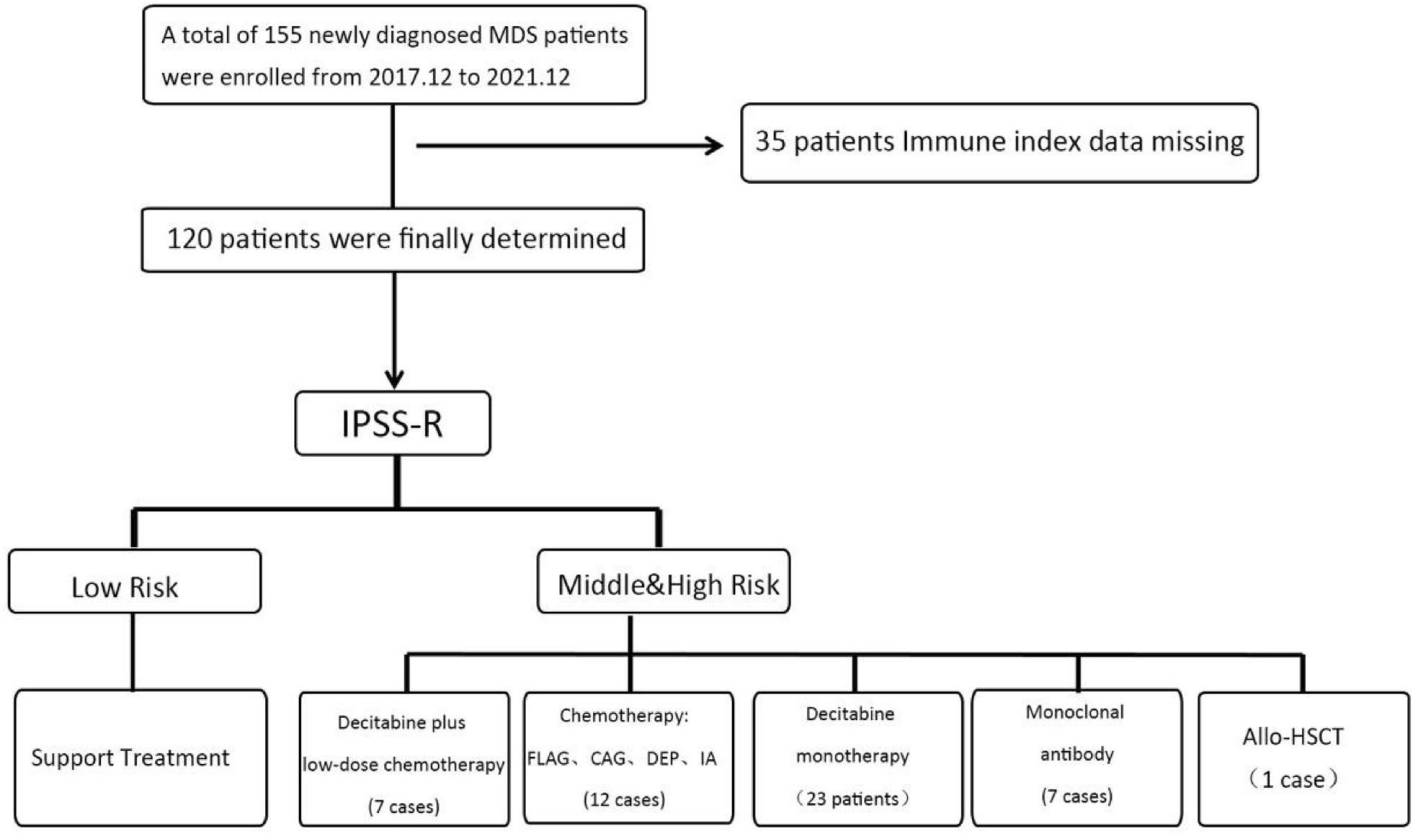
Patient enrollment determination and treatment modality

From December 2017 to December 2021, 155 newly diagnosed MDS patients in the Department of Hematology, General Hospital, Tianjin Medical University, were enrolled in the study. The IRB has approved the study and informed consents have been obtained. Following review, this article (Ethical No. IRB2022-WZ-185) conformed to “International ethical guidelines for biomedical research involving human subjects (2002)” developed by the Council for International Organizations of Medical Sciences (CIOMS) in collaboration with the World Health Organization (WHO) and the study was approved. All patients were diagnosed using morphological examination of bone marrow and peripheral blood cells, as well as karyotype analysis. The diagnosis and staging were in accordance with the MDS WHO (2008) staging criteria. The immune indices were recorded and scored according to the WPSS, IPSS, and IPSS-R scoring systems, and finally 120 patients were determined for the study. According to the IPSS-R score system and the age of patients, we should have divided them into five groups: very low-, low-, intermediate-, high-, and very high risk. However, due to the small number of enrolled patients, the variability of the components was not significant; therefore, we divided the patients into low-, intermediate-, and high-risk groups according to the treatment plan to increase the number of each group and improve the reliability of the data. The main basis of the grouping includes blood transfusion, iron removal, erythropoietin, granulocyte-macrophage colony-stimulating factor, androgen, retinoic acid, thalidomide, and other treatments. Some patients in the medium-, high-, and very high-risk groups were primarily given demethylation therapy and/or chemotherapy, including decitabine monotherapy (23 cases), decitabine plus low-dose chemotherapy (7 cases), and chemotherapy (12 cases). The chemotherapy regimens included FLAG [Fludarabine, Azacitidine, Granulocyte colony stimulating factor (G-CSF)], CAG (clarithromycin, cytarabine, G-CSF), DEP (etoposide, arubicin, dexamethasone), and IA (daunorubicin, azacitidine). Monoclonal antibody treatment (7 cases), including venecla (Bcl-2 inhibitor), programmed death-1 (PD-1) inhibitor, and CD47 monoclonal antibody. One patient underwent allogeneic hematopoietic stem cell transplantation, and the study was approved by the ethics committee of the Tianjin Medical University General Hospital. The need for informed consent was waived because the study was retrospective (Fig. 1).

### Data collection

From the patients, we the following data:, age, sex, neutrophil count, hemoglobin level, platelet count, cytopenia series, bone marrow primitive cell ratio, and karyotype. We also analyzed peripheral blood T lymphocyte subsets and complete blood counts within a week. The levels of peripheral blood T lymphocyte subsets were determined using a Beckman DxFLEX flow cytometer (California, USA).

### Statistical analysis

The immune indices included absolute counts and percentages of CD3+, CD4+, CD8+, CD56+, CD19+, IL-2, IL-4, IL-6, IL-10, tumor necrosis factor (TNF)-a, and interferon (IFN)-γ. OS was defined as the time from diagnosis to death from any cause. SPSS 26.0 software was used for statistical analysis. Receiver operating characteristic curves (ROC) and area under the curve (AUC) were used to determine the best cut-off values for survival indicated by these immune indices. Kaplan–Meier analysis was used to evaluate the OS of the patients, and the log-rank test was used for its calculations. *P*-value ≤ 0.05 was considered statistically significant.

## Results

### Determination of the cut-off value for the high and low expression groups

According to the ROC curve, the *P*-values of the ROC curve were obtained for each immune index, with the CD56+ T cell absolute count [AUC 65.6%, 95% confidence interval (CI) 0.513–0.799, *P* = 0.046] and the serum level of IL-4 (AUC 65.9%, 95% CI 0.488–0.830, *P* = 0.033) being the most significant (Table 1).

**Table 1:** ROC curve to find the cut-off value, screening indicators

Characteristic	AUC	*P*-values
CD3+ percentage	51.7	*P* = 0.830
CD8+ percentage	50.8	*P* = 0.921
CD4+ percentage	62.1	*P* = 0.122
CD56+ percentage	57.3	*P* = 0.352
CD19+ percentage	54.9	*P* = 0.529
CD3+ absolute count	64.2	*P* = 0.069
CD8+ absolute count	65.1	*P* = 0.053
CD4+ absolute count	62.4	*P* = 0.114
CD56+ absolute count	65.6	*P* = 0.046
CD19+ absolute count	56.5	*P* = 0.406
IL-2	52.1	*P* = 0.780
IL-4	65.9	*P* = 0.033
IL-6	62.6	*P* = 0.091
IL-10	51.5	*P* = 0.843
TNF-a	63.3	*P* = 0.074
IFN-γ	61.5	*P* = 0.123

### Survival analysis

**Fig. 2:**
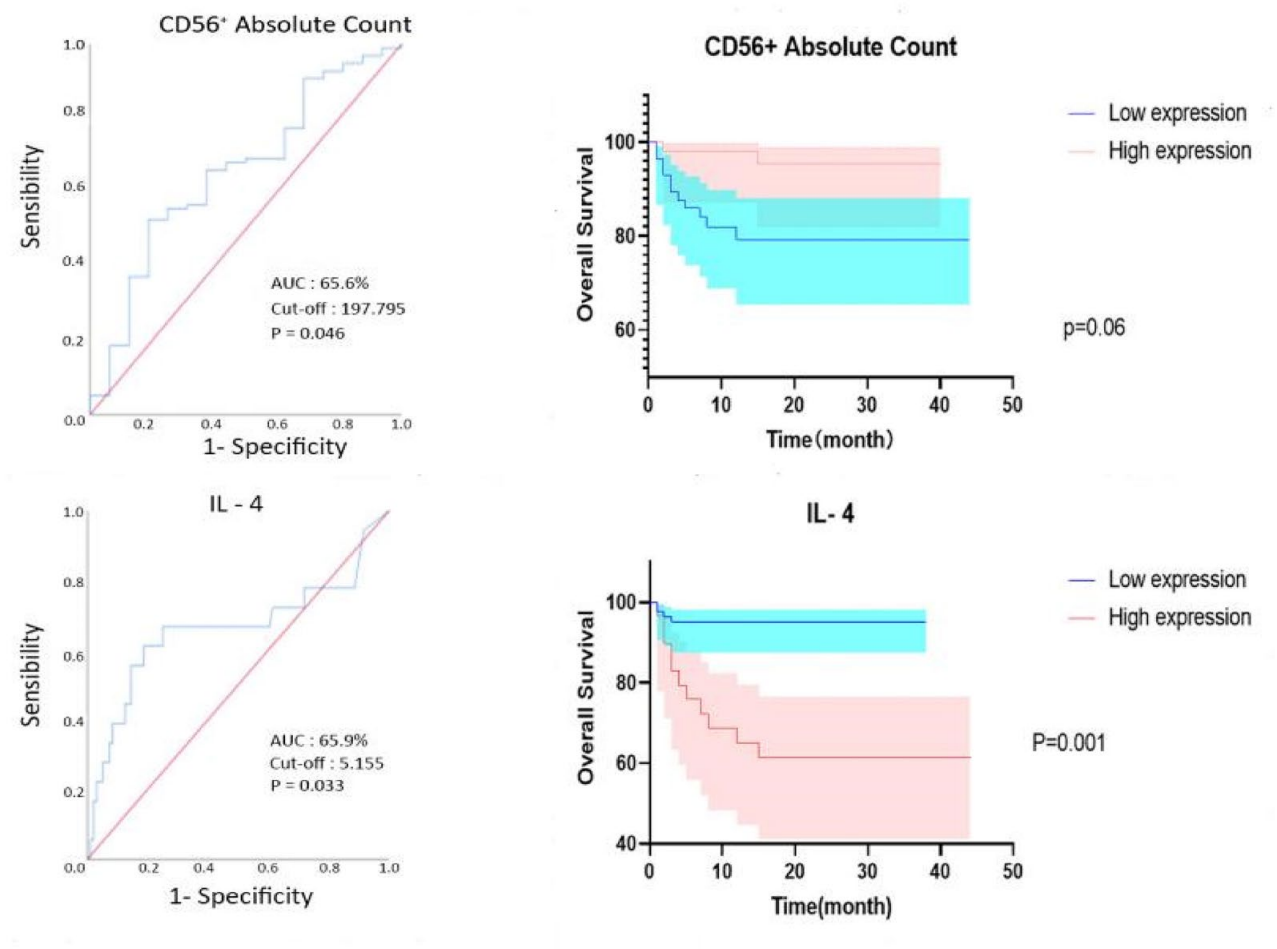
IL-4 has a good predictive value for the OS of MDS patients

The optimal cut-off value was 197.975, and patients were divided into two groups: CD56+ T cell absolute count < 197.795 and CD56+ T cell absolute count ≥ 197.795. Kaplan–Meier analysis was performed, and *P* = 0.060 was determined through log-rank test. There were no statistically significant differences between the groups. The optimal cut-off value was selected as 5.155, and patients were divided into a high expression group with IL-4 ≥ 5.155 and a low expression group with IL-4 < 5.155. Kaplan–Meier analysis was performed, and the log-rank test was used to calculate the *P* value of 0.001, indicating a statistical difference. Finally, we determined IL-4 as a meaningful indicator (Fig. 2).

### Clinical characteristics

A total of 114 newly diagnosed MDS patients with complete IL-4 data were selected, including 54 patients aged ≥ 65 years and 60 patients aged < 65 years. The study included 79 male patients and 35 female patients. There were 87 patients with a neutrophil absolute value ≥ 0.8 × 10^9^/L and 27 patients with an absolute value < 0.8 × 10^9^/L; 20 patients with hemoglobin content ≥ 100 g/L and 94 patients with hemoglobin content < 100 g/L; 36 patients with platelet count ≥ 100 × 10^9^/L and 78 patients with a platelet count < 100 × 10^9^/L; 33 patients with 0–1 cytopenia, and 81 patients with 2–3 cytopenia. The bone marrow primitive cell ratio was ≥ 2% in 79 patients and < 2 in 35 patients; 80 patients had good cytogenetic characteristics, and 34 patients had poor cytogenetic characteristics (Table 2).

**Table 2:** Characteristics at baseline of 120 newly diagnosed MDS patients

Characteristic	All cases (*n* = 114)	IL-4 < 5.155 (*n* = 85)	IL-4 > 5.155 (*n* = 29)	*P*
Age				0.038
≥ 65	54	42	12	
< 65	60	43	17	
Gender				0.083
Male	79	60	19	
Female	35	25	10	
*N* (*10^9^/L)				0.080
≥ 0.8	87	62	25	
< 0.8	27	23	4	
HB (g/L)				0.062
≥ 100	20	14	6	
< 100	94	71	23	
PLT (*10^9^/L)				0.092
≥ 100	36	24	12	
< 100	78	61	17	
Cytopenia series				
0–1	33	22	11	0.078
2–3	81	63	18	
Bone marrow primitive cell ratio (%)				0.087
≤ 2	79	56	23	
> 2	35	29	6	
Karyotype				0.095
Good	80	59	21	
Bad	34	26	8	

HB: hemoglobin , N: neutrophil, PLT: platelet, karyotype good: − Y, 11q^−^ (excellent); normal karyotype, 5q^−^, 12p^−^, 20q^−^, 5q^−^ with another karyotype abnormality attached (good); 7q^−^, + 8, + 19, i (17q), chromosomal abnormalities in other or 2 independent clones (moderate). Karyotype bad: − 7, inv(3)/t(3q)/dcl(3q), − 7/7q^−^ with one additional abnormality, 3 complex abnormalities (poor), ≥ 3 complex abnormalities (very poor)

### Analysis of prognostic factors in newly diagnosed MDS

The Cox proportional risk regression model was used to examine patient prognostic factors. After univariate regression analysis, variables with *P* < 0.05 were further included in multivariate regression analysis. Analysis of OS (showed that age ≥ 65 years (*P* = 0.045) and serum IL-4 level ≥ 5.155 (*P* = 0.013) were independent risk factors for OS (Table 3).

**Table 3:** Univariate and multivariate analysis

Factors	Univariate analysis		Multivariate analysis	
	HR (95% CI)	*P*	HR (95% CI)	*P*
Male	1.820 (0.560–5.915)	0.319		
Age ≥ 65	0.342 (0.120–0.973)	0.044	0.362 (0.141–0.977)	0.045
HB ≥ 100 g/L	0.256 (0.032–2.045)	0.199		
N ≥ 0.8*109/L	1.946 (0.525–7.208)	0.319		
PLT ≥ 100*109/L	1.092 (0.379–3.144)	0.870		
Cytopenia series ≥ 2	0.211 (0.117–1.605)	0.211		
Bone marrow primitive cell ratio ≥ 2%	0.736 (0.244–2.216)	0.586		
Karyotype: bad	0.665 (0.236–1.876)	0.441		
IL-4	0.172 (0.061–0.487)	0.001	0.237 (0.114–0.779)	0.013

HB: hemoglobin, N: neutrophil, PLT: platelet, karyotype good:  − Y, 11q^−^ (excellent); normal karyotype, 5q^−^, 12p^−^, 20q^−^, 5q^−^ with another karyotype abnormality attached (good); 7q^−^, + 8, + 19, i (17q), chromosomal abnormalities in other or 2 independent clones (moderate). Karyotype bad: − 7, inv(3)/t(3q)/dcl(3q), − 7/7q^−^ with one additional abnormality, 3 complex abnormalities (poor), ≥ 3 complex abnormalities (very poor)

### Effects of serum IL-4 on survival of MDS patients in different prognostic scoring systems

**Fig. 3:**
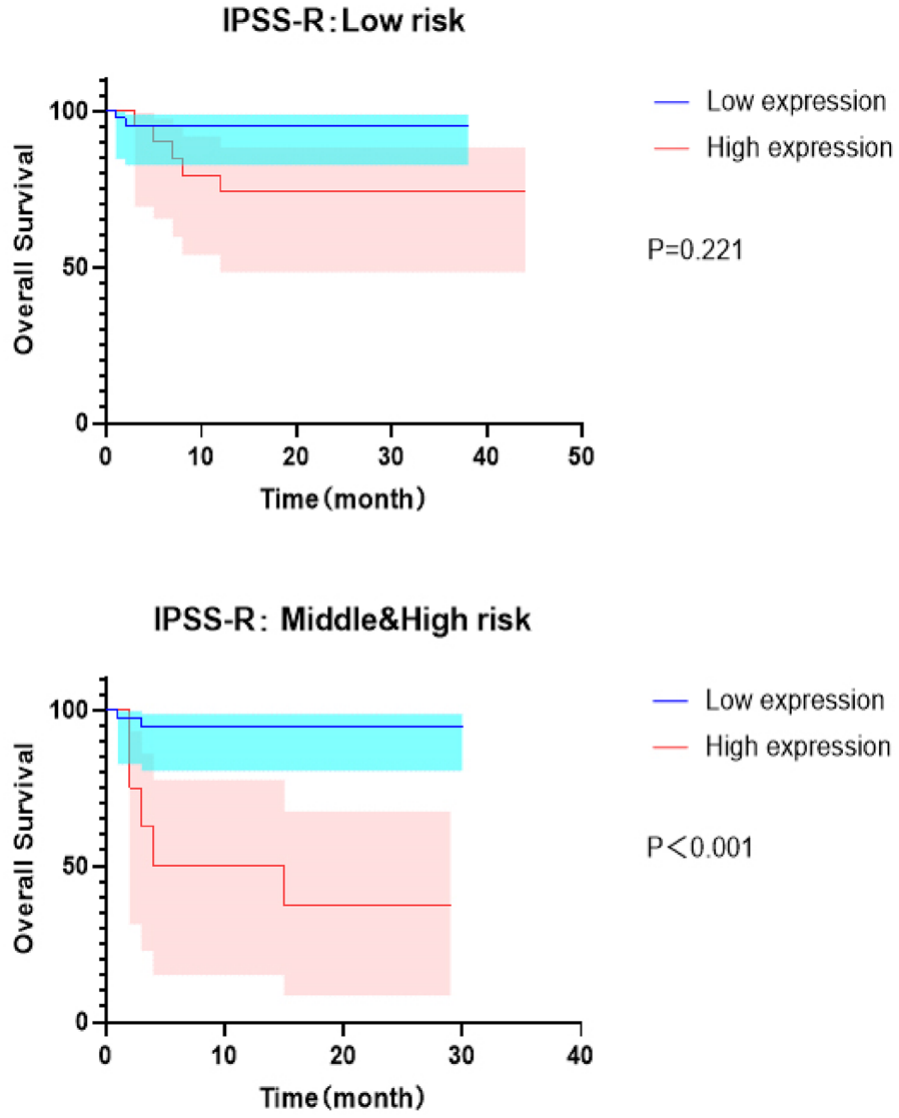
In the prognostic scoring systems IPSS-R, the serum IL-4 levels in middle and high-risk MDS patients have a predictive prognostic value

Survival curves were drawn following dividing the patients into low-risk and medium- to high-risk groups according to the WPSS, IPSS, and IPSS-R prognostic scoring systems. The results showed that in WPSS scoring system, the serum IL-4 level was an independent risk factor for medium- to high-risk patients (*P* = 0.014). In the IPSS scoring system, serum IL-4 level was an independent risk factor for low-risk and medium- to high-risk patients (*P* = 0.023, *P* < 0.001) (Additional file 1: Supplement Figure). Serum IL-4 level was an independent risk factor for medium- to high-risk patients in the IPSS-R scoring system (*P* < 0.001) (Fig. 3).

## Discussion

Immune dysregulation is closely associated with the pathogenesis of MDS. Firstly, dysregulation of T cell response and innate immune activation and its mediated bone marrow suppression are important causes of MDS, and apoptosis is often considered as a marker of low-risk MDS. In MDS, naive T cells (CD3+) exhibit shorter telomeres and significantly reduced proliferative potential [19]. MDS tumor cell immune escape occurs through dysfunctional T cell and cytokine expression and matrix changes in the hematopoietic niche [20]. Immune checkpoint inhibitors, such as PD-1 and its ligand, PD-L1, and CTLA-4, play an active role in the evasion of tumor surveillance and therapeutic resistance of MDS cells [21, 22]. Second, innate immunity consists of humoral and cellular immune mechanisms, which typically rely on Toll-like receptors (TLRS) for microbial recognition in mammals. The TLR signaling pathway leads to the activation of nuclear factor K-light chain enhancer and mitogen-activated protein kinase pathways in activated B cells, which then induce the transcription of proinflammatory cytokines [23]. In many MDS cases, TLR signaling is severely overactive due to the overexpression of activators (e.g., myeloid differentiation primary response 88, TIR Domain Containing Adaptor Protein, interleukin-1 receptor-associated kinase 4, and Tumor necrosis factor receptor-associated factor) and downregulation of repressors (e.g., miR145 and miR146a). Both MiR145 and miR146a are lost in 5Q-MDS, and these patients have a greater risk of dysplasia and transformation to acute myeloid leukemia [24–26]. Lastly, cytokines also play an important role in immune dysregulation in MDS.

In our study, we surprisingly identified immune index having significant differences between low-risk and medium- to high-risk MDS patients, and serum IL-4 showed great effects on the survival of MDS patients in different prognostic scoring systems. We suspected it as an immune index and speculate that it can be used as an important prognostic factor in patients with middle- to high-risk MDS.

Cytokines play critical roles in the circulatory system [27–32]. In lymphoid progenitor cells, IL-2 stimulates the differentiation of T cells [33], and IL-4 and IL-6 stimulate the differentiation of B cells [17]. As the most primitive hematopoietic stem cells, IL-3 drives the production of blood cell progeny [17, 21, 34]. Granulocyte–macrophage colony-stimulating factor (stimulates granulocyte–macrophage progenitors and promotes cell differentiation in erythroid pedigree lines, while G-CSF and M-CSF stimulate the most differentiated bone marrow progenitors to produce granulocytes and monocytes/macrophages, respectively [35, 36]. It is because of the existence of these hematopoietic factors that the blood system can constantly renew and self-regulate to maintain the homeostasis of the hematopoietic microenvironment. Studies have shown that the expression of at least 30 kinds of cytokines can be detected in the bone marrow and peripheral blood of MDS patients, among which the increased levels of TNF-a, IFN-γ, TGF-β, IL-6 and IL-8 directly reflect the serious dysregulation of inflammatory signal conduction and bone marrow differentiation [11, 37–39]. The levels of cytokines in the innate and adaptive immune responses are different, and the levels of cytokines in MDS patients with different risks are also significantly different [7]. In innate immunity, since NK cells can mediate the cytotoxicity of bone marrow precursor cells, macrophages are more cytotoxic to bone marrow precursor cells in low-risk MDS patients. As a result, higher frequencies of NK cells and macrophages were observed in low-risk MDS patients than in high-risk MDS patients, and DCS expression was low in both low-risk and medium- to high-risk MDS patients. In the adaptive immune response, the bone marrow mesenchymal cells of MDS patients lose the potential to differentiate into B-cell progenitors, so the expression of B-cell-related factors and Treg cells is reduced. However, cytotoxic CD8+ T cells, NK T cells, and Th17 cells are all highly expressed in low-risk MDS patients and poorly expressed in medium- and high-risk MDS patients [40, 41]. In terms of pathogenesis, low-risk MDS is usually characterized by elevated apoptosis, whereas high-risk MDS has been shown to be associated with more aggressive clonal expansion [4]. IFN-γ and IL-6 levels are closely related to the induction of apoptosis in MDS patients’ BM; thus, higher secretion of IFN-γ and IL-6 is usually associated with low-risk MDS [42]. However, immunosuppressive cytokines, such as IL-10, are more strongly secreted in high-risk MDS patients [33, 43].

IL-4, IL-3, IL-5, IL-9, IL-13, and transcription factor GATA3 belong to the Th2 cytokine family, which plays important immunomodulatory roles and have corresponding effects on a variety of immune cells. These include B cells, eosinophils, basophils, monocytes, fibroblasts, endothelial cells, airway epithelial cells, smooth muscle cells, and keratinocytes. Th2 cells are one of the major sources of IL-4 due to their ability to amplify antigens [44]. Other sources of IL-4 include follicle-helper Th (Tfh) cells located in lymph node B-cell follicles, a unique subset of T cells called NKT cells, and certain innate immune cells, such as mast cells, basophils, and eosinophils [11, 44, 45]. There are two main types of receptors that specifically bind to interleukin-4. Type I receptors are composed of receptors that only bind to interleukin-4, while type II receptors are composed of receptors that can bind to both interleukin-4 and interleukin-13 [11]. Many reports have shown that IL-4 plays an important role in mediating inflammatory reactions and can play an important role in type I allergic reactions. Interleukin-4 polymorphism is widely correlated with rheumatoid arthritis/atopic dermatitis/allergic rhinitis [27, 46, 47] and other diseases. In addition, IL-4 can also cause capillary leakage syndrome and IL-4 associated hematuria and gastroduodenal lesions; however, there are few studies on the mechanism of IL-4 in MDS patients and its impact on prognosis. We hope that future clinical studies will enrich the content of this review.

Meanwhile, we found that age ≥ 65 is also an important risk factor for the prognosis of MDS patients. Reinhard Stauder et al*.* showed that anemia is particularly common in the elderly and is a key indicator of various reactive and clonal diseases. Many underlying diseases with anemia as the main presentation, such as myelodysplastic syndrome (MDS), develop preferentially in the elderly [48, 49]. Due to advanced age, comorbidities, and a lack of histocompatible donors, patients with MDS have lost the opportunity for allogeneic hematopoietic stem cell transplantation [50]. MDS is a chronic disease. Comorbidities must be considered in the elderly because they are often accompanied by other basic diseases and present with more clinical symptoms, resulting in a worse prognosis.

In the diagnosis of MDS, we often monitor a series of immune indicators, but few people pay attention to the correlation between the expression level of these immune indicators and the survival of low-risk and medium- to high-risk MDS patients. Considering the important role of IL-4 in the diagnosis of MDS, we speculate that IL-4 can be used as an important part of the prognostic scoring system to predict the survival of MDS patients. However, our study has some limitations. Our study was a retrospective study on clinical case data, rather than a prospective study, which has certain limitations. Moreover, due to the limited follow-up time and the small number of people screened in the group, there may be bias. There is a 30% chance that MDS will progress to leukemia [51, 52]; therefore, studies often use conversion to leukemia as an important clinical parameter to determine the prognosis of MDS. In our study, the small number of patients enrolled and the fact that most patients were lost to follow-up after treatment makes it more difficult to study patients with conversion to leukemia; therefore, this important indicator is not reflected in this study. In 2022, an updated prognostic scoring system for MDS was proposed called the new Molecular International Prognostic Scoring System (IPSS-M) [53, 54]. In this new scoring system, patients are stratified more finely in terms of risk from a molecular perspective [55]. In this study, we should have also studied the patients in relation to the IPSS-M, but we were unable to perform an IPSS-M-related analysis because the patients were not tested at enrollment and because of the limitations of the current testing methods, and a large number of clinical trials are needed to confirm our hypothesis.

## Conclusion

According to existing studies, it is not difficult to find that immune factors play a crucial role in the pathogenesis of MDS and are directly related to patient prognosis. However, the immune index, which is an important factor, has not been studied in various international prognostic scoring systems. We conducted a retrospective study to discuss the possibility of incorporating immune markers into a prognostic scoring system to assess patient survival. However, there were limitations and biases in our study. We hope that more people will pay attention to this part of the content in the future, and verify our conjecture through a large number of subsequent clinical trials.

## Supplementary Information

**Additional file 1. Fig. S1**: Patients were divided into five groups according to the IPSS-R scoring system, and a comparison of the differences between groups was performed for each immunological index. *P *< 0.05 was statistically significant. **Fig. S2**: In the other two scoring systems, WPSS and IPSS, the serum IL-4 levels in middle and high-risk MDS patients have a predictive prognostic value.

## Data Availability

All data generated or analyzed during this study are included in this published article.

## Abbreviations

AUC: Area under the curve
CD: Cluster of differentiation
CIOMS: Council for International Organizations of Medical Sciences
G-CSF: Granulocyte colony stimulating factor
IFN: Interferon
IL: Interleukin
IPSS: World Health Organizational prognostic scoring system
IPSS: International prostate symptom score
IPSS-R: Revised international prostate symptom score
MDS: Myelodysplastic syndrome
OS: Overall survival
PD-1: Programmed death-1
ROC: Receiver operating characteristic curves
TNF: Tumor necrosis factor
TLRs: Toll-like receptors
WHO: World Health Organization
