# Supplementary material for: The value of serum IL-4 to predict the survival of MDS patients
Source: Eur J Med Res. 2023 Jan 4;28:7. doi: 10.1186/s40001-022-00948-w (PMC9811803; doi:10.1186/s40001-022-00948-w)
Supplement: Supplementary file 1 — Additional file 1. Fig. S1: Patients were divided into five groups according to the IPSS-R scoring system, and a comparison of the differences between groups was performed for each immunological index. P < 0.05 was statistically significant. Fig. S2: In the other two scoring systems, WPSS and IPSS, the serum IL-4 levels in middle and high-risk MDS patients have a predictive prognostic value. [file 40001_2022_948_MOESM1_ESM.docx]

Supplementary materials


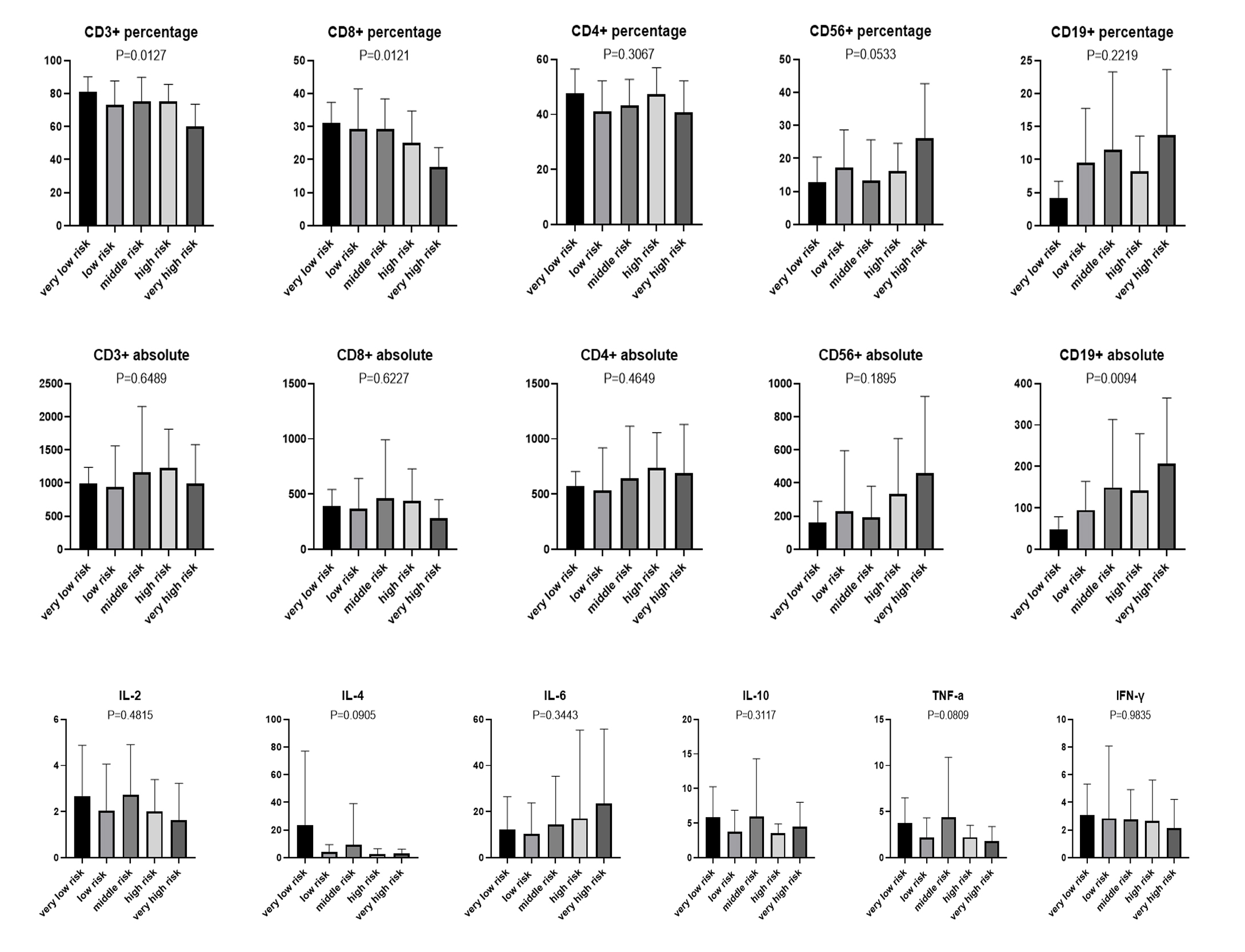


*Figure 1.*Patients were divided into five groups according to the IPSS-R scoring system, and a comparison of the differences between groups was performed for each immunological index. p<0.05 was statistically significant.


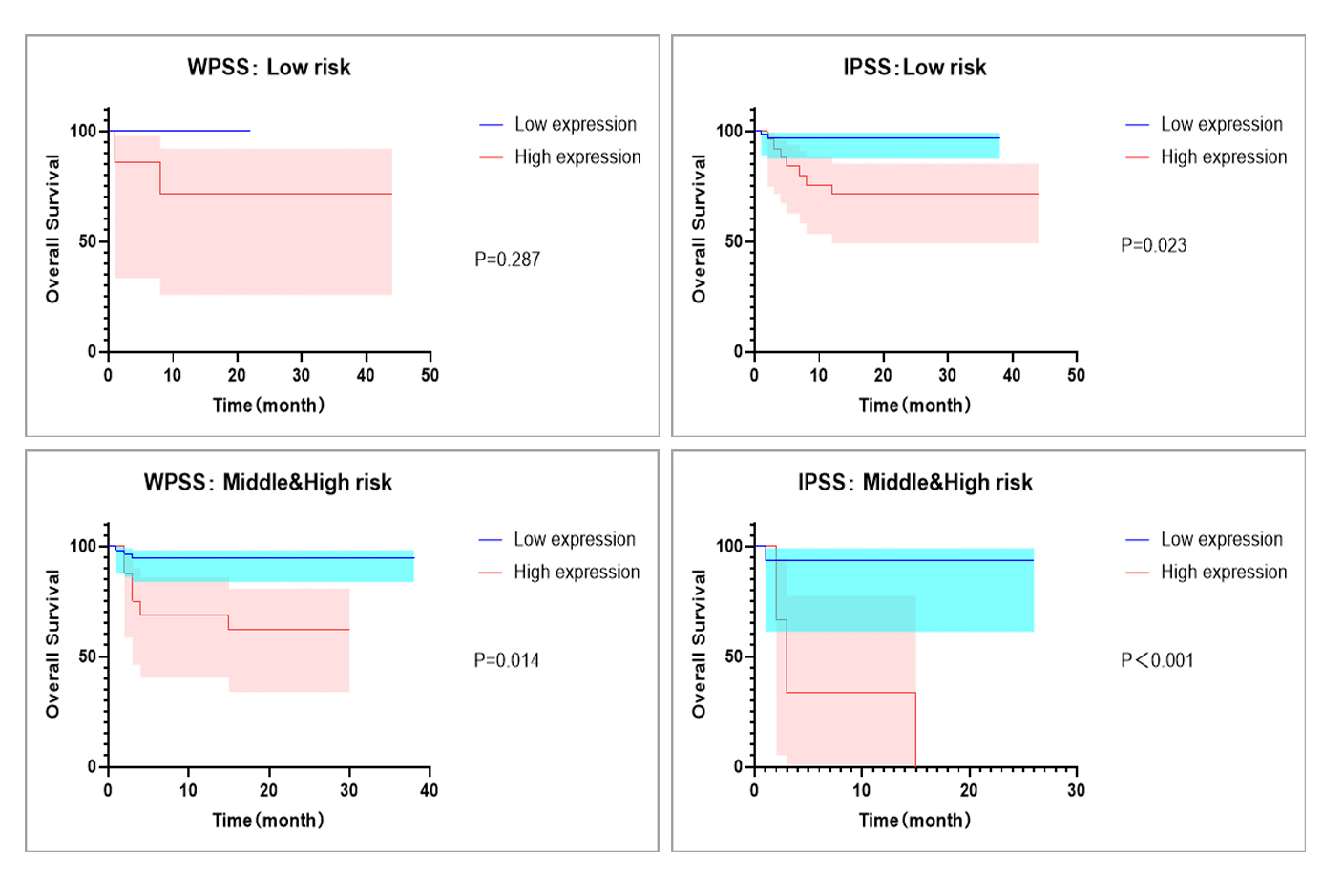


Figure 2. In the other two scoring systems, WPSS and IPSS, the serum IL-4 levels in middle and high-risk MDS patients have a predictive prognostic value
